# “I would not want the mechanic to direct me to an engine repair manual”: a qualitative analysis of provider perspectives on low-intensity treatments for patients on waiting lists

**DOI:** 10.1186/s12888-023-05055-6

**Published:** 2023-08-17

**Authors:** Allison Peipert, Sydney Adams, Lorenzo Lorenzo-Luaces

**Affiliations:** Department of Psychological and Brain Sciences, Indiana University Bloomington, 1101 E 10th Street, Bloomington, IN 47405 USA

**Keywords:** Psychotherapy, Digital mental health, Self-help, Waiting lists

## Abstract

**Background:**

Low-intensity treatments (LITs), such as bibliotherapy or online self-help, have the potential to reach more individuals than traditional face-to-face care by circumventing many of the common barriers to mental health treatment. Despite substantial research evidence supporting their usability and efficacy across several clinical presentations, prior work suggests that mental health providers rarely recommend LITs for patients waiting for treatment.

**Methods:**

The present study analyzed provider open responses to a prompt asking about perceived barriers, thoughts, and comments related to additional treatment resources for patients on treatment waiting lists. We surveyed 141 practicing mental health providers, 65 of whom responded to an open text box with additional thoughts on using LITs for patients on treatment waiting lists. Responses were qualitatively coded using a thematic coding process.

**Results:**

Qualitative outcomes yielded 11 codes: patient appropriateness, research evidence, feasibility, patient barriers, liability, patient personal contact, additional resources, positive attitudes, trust in programs, systemic problems, and downplaying distress.

**Conclusions:**

Results suggest providers are predominantly concerned about the potential of suggesting a LIT that would be ultimately inappropriate for their patient due to a lack of assessment of the patient’s needs. Furthermore, providers noted ambiguity around the legal and ethical liability of recommending a LIT to someone who may not yet be a patient. Guidelines and standards for recommending LITs to patients on treatment waiting lists may help address ambiguity regarding their use in routine care.

## Background

The demand for mental health services has increased significantly in recent years, with rates of common mental disorders on the rise [1] and a shortage of available professional providers to meet the growing need [2, 3]. Reported wait times for mental health appointments prior to the COVID-19 pandemic are highly variable based on a number of factors, including country income, specialty vs. non-specialty setting, rural vs. urban location. Some studies report wait times as brief as 3-weeks and others report a 3-month average wait time [4, 5]. Moreover, the COVID-19 pandemic has increased mental health needs worldwide [6]. Since the pandemic, providers have experienced an increase in estimated waiting time for patients seeking mental health services [7].

Waiting for mental health treatment is associated with numerous negative outcomes. First, waiting for mental health treatment is associated with increased symptom severity [8]. Second, longer waiting times are associated with decreased engagement during treatment [9, 10]. Third, waiting time may also be associated with decreased probability of improvement in treatment [11]. Finally, wait time may be associated with an increased risk for drop out prior to treatment initiation and during treatment [9]. In qualitative research, patients also report waiting time for mental health care as a barrier for accessing treatment, as well as negative psychological and behavioral outcomes while on treatment waiting lists [12].

One proposed strategy for addressing the burden of mental health care and barriers to face-to-face treatment are low-intensity treatments (LITs). LITs for common mental disorders can include print media resources (e.g., bibliotherapy) and internet-based resources (e.g., apps, internet-based cognitive behavioral therapy [13]). LITs can be used with guidance from a professional or paraprofessional (i.e., guided) or self-guided by the individual user (i.e., unguided). Face-to-face psychotherapy (e.g., cognitive behavioral therapy) is regarded as the standard for common mental disorders like depression, anxiety, or insomnia [14–16]. However, research has shown that both guided and unguided LITs can be more effective than care as usual or waitlist controls [17–20], and guided LITs can be equally as effective as face-to-face care [21]. Because LITs are low-cost interventions with the potential to be accessed by numerous patients for whom traditional face-to-face interventions are not accessed, LITs appear to have a greater potential to reduce the burden of untreated common mental disorders.

Research on the use of LITs specifically for patients waiting for treatment is limited. Preliminary studies, however, support the feasibility of implementing LITs, such as mental health apps, for treatment waiting lists. In one study by Levin and colleagues (2022) students on a college counseling center waiting list were randomized to receive a mindfulness app while on the waiting list or no intervention (i.e., waiting list only). Though power was limited, the researchers found potential efficacy of the app intervention, such that large effect sizes were found from pre- to post-intervention for depression and general distress [22]. More importantly, participants reported high satisfaction and moderate usage with the app during the waitlist period. Similarly, a study by Hentati and colleagues (2022) provided participants placed on a waiting list for psychiatric care with a digital problem-solving app. Most participants rated the intervention as credible and usable, with engagement levels comparable to other self-guided interventions [23].

In a previous study, Peipert et al. (2022) surveyed 141 providers regarding their waiting list practices and attitudes towards LITs for patients awaiting treatment [7]. Most (69%) of the included providers maintain a treatment waiting list. Among those that reported not maintaining a waiting list, nearly half reported scheduling patients in the “distant future,” such as 2–3 months in the future. As such, 83% of the sample had opportunity to suggest LIT usage for patients prior to them being seen for an in-person session. Despite this opportunity, few (< 20%) reported currently recommending LITs for patients awaiting treatment. Interestingly, attitudes towards LITs were mixed-to-favorable, suggesting a need to understand the barriers to LIT recommendation. To better understand attitudes towards LIT use during waiting lists, we used qualitative methods to explore provider perspectives of LIT use for patients on treatment waiting lists.

## Methods

### Participants

We surveyed practicing mental health care providers on their attitudes towards LITs, such as guided and unguided bibliotherapy and online self-help [7]. Participants were recruited via social media (i.e., Twitter) and through emails posted to professional organization listservs, specifically the American Psychological Association Division 29: Society for the Advancement of Psychotherapy Research, and Association for Behavioral and Cognitive Therapies. Participants were considered eligible to participate if they were currently a licensed mental health care professional providing at least 1 h of clinical services per week.

### Data

The survey was advertised as a survey on “waiting lists and possible resources” and included questions related to waiting list practices, attitudes towards LITs, opportunity to receive additional information on LITs, and an open response prompt to provide additional thoughts on using LITs for treatment waiting lists (“This survey is designed to be brief, but we are very interested in your perceptions of these resources. We are especially interested in potential barriers to use. Please provide any additional thoughts or comments you have about resources or treatment modalities for clinical practice waiting list.”). The survey and data can be found in the online materials [24]. Study procedures were approved by the institution’s Human Subjects & Institutional Review Board.

### Qualitative analysis plan

We used thematic analysis to identify patterns of meaning, or themes, within the data [25]. The coding process employed two independent coders (AP & SA) who analyzed the data using an inductive approach to generate initial codes. Each coder read through the data and generated a preliminary codebook that captured the most salient concepts from the data. The two coders then compared and discussed discrepancies to revise and develop a consensus codebook that integrated their individual codebooks. Any disagreements were resolved through discussion until full coding consensus was achieved. Once a consensus codebook was established, both coders independently applied the codes to the entire dataset. They discussed any discrepancies until they achieved consensus on the final set of themes.

## Results

141 providers responded to the survey, 65 of whom completed the optional open response question. The open response responders were primarily female and non-Hispanic White. Participants’ average age was about 41-years. Most participants who completed the survey had a Ph.D. and prescribed to an orientation related to cognitive-behavioral therapy (CBT; e.g., cognitive, third-wave behavioral). Over half of survey completers were employed in a private practice setting with an average of 12.6 years of clinical experience (SD = 11.9) Full demographic and clinical variables from the total survey sample (N = 141) are reported in Peipert et al. (2022). See Table 1 for full demographic information of those who responded to the open response question.

**Table 1:** Demographic and clinical variables of 65 providers who responded to an open response question in a survey of waiting lists and low intensity treatments

		N (%), mean	SD
Age		41.1	10.3
Gender			
	Female	44 (67.7%)	
	Male	19 (29.2%)	
	Nonbinary	2 (3.1%)	
Race/ethnicity			
	Non-Hispanic White	53 (81.5%)	
	Non-Hispanic Black	1 (1.5%)	
	Hispanic	5 (7.7%)	
	Asian	3 (4.6%)	
	AIAN, MENA, NHPI, Other	1 (1.5%)	
	Multiracial	2 (3.1%)	
Education			
	MA	6 (9.2%)	
	PhD	54 (83.0%)	
	PsyD	5 (7.7%)	
Private Practice (vs. not)		41 (63.1%)	
Theoretical Orientation (CBT vs. not)		58 (89.2%)	
Clinical experience (years)		12.6	11.9
Clinical hours/week		19.7	10.3

*Note*. N = 65, SD = standard deviation, AIAN = American Indian or Alaska Native, MENA = Middle Eastern or North African, NHPI = Native Hawaiian or Pacific Islander, MA = master’s degree, PhD = doctor of philosophy, PsyD = doctor of psychology

Thematic coding yielded 11 themes across the 65 open responses: patient appropriateness, feasibility, research evidence and efficacy, patient barriers, liability, patient personal contact, additional resources, positive attitudes, trust in programs, systemic problems, and downplaying distress. The themes and example quotes are presented in Table 2.

**Table 2:** Theme label, definition, and example quotes from 65 open responses about recommending LITs for patients on treatment waiting lists

Theme	Frequency Mentioned	Definition	Example Quote
Patient appropriateness	27	Factors that affect whether a patient is or is not appropriate for using a LIT while on a treatment waiting list (e.g., need for evaluation, level of risk, diagnosis)	I don’t have time to evaluate what prospective patients might need, and wouldn’t want to recommend resources to them without knowing them better.
Feasibility	20	Aspects that influence the logistics of offering LITs to patients (e.g., time, money, staff, etc.)	It is difficult to recommend something to a client that I have not personally vetted; so the biggest barrier is finding the time to learn about the available resources.
Research evidence and efficacy	18	How effective a LIT would be for patients on a waiting list and the availability of research evidence to support the use of LITs on patient waiting lists.	One problem is the lack of good, evidence-based self-help books for PTSD or trauma. Most of what is out there is pop psychology and has not been tested.
Patient barriers	15	Barriers at the patient level that would prevent providers from recommending LITs for patients on a treatment waiting list (e.g., money, access to resources, literacy level).	For the families I work with, there are financial barriers to purchasing books or apps. Additionally, I work with many children with limited reading and cognitive abilities and many parents for whom English is not their dominant language and may have limited reading abilities
Liability	9	Legal or ethical liability related to LIT use with patients	I am reluctant to provide any clinical care to someone who is not officially my patient. I think that is ethically a little sticky.
Patient personal contact	8	Reference to contact between patients and providers in the context of mental health treatment	Many individuals want an actual person and not interested in materials or apps
Additional resources	11	Alternative resources suggested by providers for patients waiting for treatment (e.g., alternative providers, online resources, relaxation techniques)	It’s important to be able to have a range of things to offer which meet the needs or wants of the client. Sometimes I recommend podcasts or blogs, or free online guided relaxation exercises for example
Positive attitudes	18	Reflections of positive aspects of LITs and their use by patients	Self help books are readily accessible
Trust in programs	6	Attitudes towards LIT programs that reflect a level of or lack of trust (e.g., security, intentions)	I am hesitant to recommend online services [because] I am uncertain about security and setting up vulnerable people to businesses that have nonclinical motives.
Systemic problems	4	Reference to problems at a larger systemic level that influence providers’ attitudes towards or ability to recommend LITs (e.g., working conditions, access to resources)	Though the working conditions and pay are abysmal at best, I am passionate [about] community mental health and am quite frankly tired of seeing my clients and community get the leftovers of the system.
Downplaying distress	2	Concerns around patients feeling dismissed by being offered services that are considered less than their needs require.	My concern in recommending these options is that the caller/would-be client may perceive the recommendations as downplaying the significance of their distress.

### Patient appropriateness

A frequent theme mentioned in the open responses was concerns about whether a patient may be appropriate for using LITs while on a waiting list. This theme appeared to reflect two main concerns: (1) lacking information available on the patient to recommend an intervention at the time of being placed on a waiting list, and (2) a perceived mismatch between the client population served with those for whom LITs would be helpful. Responders noted a perceived lack of appropriateness for LIT use given they have not had time to do a proper assessment of the patient prior to them being placed on the waiting list. Therefore, the clinician would not know what LIT to recommend which would be appropriate for that patient. Other factors associated with patient appropriateness included level of severity, of perceived risk, a perceived lack of available LITs for their target population (e.g., couples, eating disorders, multiple comorbidities), and individual patient’s level of motivation.

### Feasibility

Providers identified barriers associated with the feasibility of recommending LITs for patients on a waiting list. These included a general lack of knowledge of available LITs, feeling overwhelmed by the number of LITs available, and the effort and time required to acquire the required level of knowledge to integrate LITs into waiting list practices. Participants also discussed confusion around billing and compensation for LITs, and a lack of insurance coverage. At an administration level, participants mentioned a lack of system support (“…the clinic has not been open to it for reasons that are unclear to me”), a lack of personnel dedicated to LIT delivery, and lack of training around integrating LITs into clinical practice. Finally, one participant noted the requirement to generate revenue in order to continue providing services may hinder them from recommending LITs for patients waiting for treatment: “… I am running a business. Businesses have to generate revenue to survive. I don’t feel a responsibility to advertise other businesses, such as online guided programs and bibliotherapy coaches, to people who call my business!”

### Research evidence and efficacy

Participants noted barriers related to a lack of strong research evidence for LITs, or beliefs around LITs not being effective for their patients. One noted, “From a risk management perspective, I see no evidence thus far to demonstrate the efficacy of such programs. These programs may give the clinician a false sense of doing good for their patient on a waitlist when in fact they may be doing harm.” Another wrote, “[Apps] tend to be underresearched and overhyped.” Some of these comments were specific to the guidance or coaches used in guided LITs (“I would not refer to a coach as they are untested and inconsistent”), while others pointed out that efficacy may vary depending on the individual and their needs (e.g., diagnosis, comorbidities, level of severity). Conversely, a few individuals noted there was sufficient evidence to support the use of LITs in clinical practice (“I have suggested to our clinic having a “wait-list” intervention program (1–3 sessions with suggestions of apps, books, and other resources) since there is evidence to support this type of program”). One individual noted an interest in knowing more about research on LIT efficacy and uptake.

### Patient barriers

Providers noted a variety of barriers that patients face which prevent them from recommending LITs. The most commonly noted barriers were access to resources such as internet and computer for online programs, and money to pay for an app or book. Furthermore, some providers believed patients may be limited in their abilities, such as those with intellectual disabilities, visual/hearing impairments, low literacy levels, and a lack of knowledge or familiarization with technology that may prevent them from comfortably navigating a phone- or computer-based program. Many providers noted patients experience a lack of privacy outside of the therapy office which may inhibit patients’ ability to participate with a LIT. Another provider noted that some patients are limited to only services which will be reimbursed by insurance, which prevents them from recommending LITs. A few providers noted lack of motivation or willingness to complete LITs as a barrier for their patients. Language was also mentioned as a patient barrier, such that most online interventions and guided interventions are not available or accessible in a non-English language for their patients.

### Liability

Providers questioned the legal and ethical liability of offering a LIT for patients on a waiting list. In the case of providing guidance for those using a LIT, it was unclear to some providers the extent to which the recipient would be officially a “patient” rather than someone on the waiting list who is not yet a patient in their clinic. One participant wrote, “I am reluctant to provide any clinical care to someone who is not officially my patient. I think that is ethically a little sticky.” Providers were also concerned about using LITs with high-risk patients, “liability concerns – if we provide any “between” support, are we then liable for the patient if something happens before they begin [our] treatment?”

### Patient personal contact

Providers expressed that personal contact was an important factor for patients when considering use of LITs. Some expressed that patients need personal contact to be accountable and engaged in a LIT. Others mentioned that patients have varying needs when it comes to personal contact, where some require personal contact and others may simply prefer it. One provider noted that patients would not use LITs because of their interest in developing a therapeutic relationship, which would be more effective for their treatment overall. Finally, one provider mentioned LITs may be a useful adjunct to the therapy relationship, though not a standalone intervention.

### Additional resources

Providers highlighted resources they recommend to patients when waiting for treatment. Many included the assumption that patients will seek out a different provider when on the waiting list “and not stay on wait-list.” Other resources that providers mentioned include websites, books, podcasts, blogs, and relaxation exercises. Though some of these may overlap with LITs, most were not considered to be treatment-related, but rather psychoeducational. One provider also mentioned accessing books available through public libraries for individuals who face financial barriers.

### Positive attitudes

Providers expressed positive attitudes towards LITs in their open responses. These included statements related to using or recommending LITs already, or an interest in changing behavior to recommend LITs for patients waiting for treatment, “I would be willing to use most of the options listed above.” Others expressed the belief that these options are effective, helpful, and accessible to patients, and that LITs would be useful specifically for those waiting for treatment. One provider theorized that starting a LIT prior to face-to-face treatment may lead to faster improvement overall, “…perhaps I should be willing to consider giving my couples resources who are waiting to get in to see me to see if that would help them progress faster when they start.” Providers expressed a common goal towards increasing access to care and decreasing wait times, and the possibility that research could lead to insurance coverage for LITs.

### Trust in programs

Some provider’s comments suggested a hesitancy to use LITs given that they have not personally vetted these resources. A couple comments highlighted uncertainty around the security and financial motives of LITs, “I am hesitant to recommend online services [because] I am uncertain about security and setting up vulnerable people to businesses that have nonclinical motives.” Another comment highlighted distrust towards the coaches involved in guided intervention. Finally, one comment included beliefs that most LITs do not include all aspects of a treatment or address all presenting problems, but instead use a “superficial approach.”

### Systemic problems

A few responses commented on systemic problems that prevent people from seeking or accessing quality mental health care. One provider noted how clinical practice procedures can erect barriers that cause clients to wait for treatment or drop out prior to accessing care, such as requiring a phone screen. Another provider discussed the systemic issues associated with community-based care, including high caseloads, low pay, and high turnover rates, which ultimately impact the quality of patient care. Furthermore, this provider added that mental health care systems would use LITs as replacements for therapy to reduce overall expenses. “We serve some of the most vulnerable. They deserve every bit the same high quality care offered to commercially insured or private pay clients.” Overall, this responder highlighted how LIT use may further increase mental health disparities rather than increase equity because the root causes of systemic barriers remain unaddressed while LITs may become more common for those who have higher-level needs in low-resource settings. “So, when I think about ways to manage wait lists, replacing even temporarily therapy with AI, apps, or even a peer support group just doesn’t cut it. Is it better than nothing? Maybe. Is it how we should be designing our systems or where we should be dedicating resources? Absolutely not. It just doesn’t meet the need.”

### Downplaying distress

One provider noted concerns around patients feeling dismissed by being offered a service that was less intense than face-to-face psychotherapy.

## Discussion

The results of the thematic analysis highlight key barriers to LIT implementation for patients placed on treatment waiting lists. The most commonly noted barriers include an understanding (or lack thereof) of patients who may or may not be appropriate for LIT use, provider feasibility for providing LITs (e.g., time, access to information), knowledge of research and beliefs around the efficacy of the intervention, patient barriers for accessing LITs (e.g., language, access to resources), and legal or ethical liability to providers when recommending resources to patients awaiting treatment.

Several limitations of the current data are worth considering. First, we qualitatively analyzed open responses collected from a survey rather than pursuing more thorough qualitative methods such as focus groups or interviews. Additionally, our clinicians may not be representative of the population of clinicians who could use LITs in their practice, placing limitations on the generalizability of the barriers identified. For example, 63% of providers who responded worked in private practice settings, rather than hospital clinics or academic medical settings. However, a strength of the study is that other clinical characteristics (e.g., gender, race, ethnicity) are relatively representative of data on practicing psychologists [26]. Finally, clinicians differed on how much they knew about, and had tried, LITs, thus not all barriers that were identified could represent barriers to changing practice. Nonetheless, a strength of the study is that most clinicians currently did not use LITs in their practice, thus giving us a window into perceived barriers related to implementing LITs for the first time.

Many of the identified barriers align with recent research on the implementation and dissemination of other low-resource interventions. A recent paper by Woodard et al. (2023) highlighted barriers identified by nonspecialist providers (NSPs; e.g., college graduates) delivering a brief behavioral treatment for depression [27]. The results suggest that barriers to implementation of behavioral treatments by NSPs included its credibility, feasibility, and appropriateness, including questions about the adequacy of the dissemination method for the population, the need for more intensive services, and incongruences between perceived need for more intensive services. Our results overlap to suggest that providers make a distinction between individuals who are and are not suitable to have an LIT recommended. Prior work suggests that individuals are *deemed* more suitable for LITs when they are young, less severely ill, and have greater psychosocial resources [28–30]. Interestingly, large-scale studies do not reliably support these perceptions. For example, severity does not predict a poor response in LITs more than it does in higher-intensity treatments [31]. Additionally, younger age is associated with engagement and positive attitudes towards digital interventions, but older adults have higher retention rates in LITs than younger individuals [32]. Thus, clinician judgements of predictors of LIT engagement do not align with the literature.

Moreover, it seems that individuals perceive a mismatch between the patients’ needs for intensive services and the very nature of LITs, including the possibility that the very act of offering an LIT could be seen as downplaying the patient’s distress. For instance, one provider noted, “If I took my car in for repairs, I would not want the mechanic to direct me to an engine repair manual,” implying that patients who receive a LIT recommendation from a mental health provider may feel they are being passed up on the services they came for and recommended to fix their problems on their own. Many providers perceived that currently available LITs were not appropriate for specific populations (e.g., families, couples), problems they regularly encounter (e.g., eating disorders), or individuals who may have difficulty independently accessing or understanding LIT materials. These barriers highlight areas of improvement for the field, which has largely focused on LITs for depression, despite the promise of LITs for treating other clinical symptoms. One specific aspect of treatment that was mentioned is the importance of personal contact (see Woodard et al., 2023). While prior work suggests that personal contact increases the efficacy of and engagement with LITs, there is also work to suggest that individual patients enjoy the flexibility afforded by LITs even as they sometimes desire more contact [33–35]. Additionally, though providers may *perceive* LITs as a barrier to generating rapport with patients, other studies have found relationships in guided iCBT to be non-inferior to client-therapist relationships [36].

The providers in our study also highlighted barriers that have received less attention in existing research on LITs. Specifically, providers voiced concerns around the trust in mental health companies or apps which make a profit by providing a resource which may or may not have been vigorously tested. One notable barrier highlighted was systemic problems related to the quality of care delivered in community-based settings. Some providers appear to be concerned that recommending LITs is tantamount to delivering lower quality care. These providers were not necessarily concerned about the impact on current care, but rather that there would be system-level consequences to LIT implementation that would further exacerbate inequities in quality mental health care access. Future work should address framing of LITs, so they are seen as a supplement to high quality care rather than a replacement. Previous work discusses this differentiation (see Williams & Martinez, 2008 [37]), such that the hierarchical framing of low intensity compared to higher intensity care implies LITs are of a lower quality. This is not necessarily true, as many LITs and forms of self-help show similar effectiveness to face-to-face treatments. However, the concern may instead be that someone who will only benefit from a higher intensity treatment will be required to access LITs instead. Additional research is needed in this area to determine who is best suited for different levels of intensity in psychotherapy. As noted previously, clinical judgement can be misaligned with research findings in terms of patient appropriateness for LITs. Rather, we suggest the framing of LITs in clinical care focus on meeting patient preferences (e.g., not making stepped care a requirement for face-to-face care), increasing optimism around LITs, and other well-researched change mechanisms that can improve engagement in LITs. Finally, some providers noted concern about the legal liabilities incurred if recommending an intervention to someone who is not yet a patient, especially if one has not done an evaluation. Guidelines on the recommendation of LITs in the context of waiting lists may serve to decrease attitudinal barriers to their uptake.

Our qualitative analyses identified perceived barriers to implementing LITs as well as individual differences in potential system barriers. Future work should explore implementation strategies to increase of LITs in routine care. Woodard et al., for example, noted that doubts regarding credibility and efficacy can be alleviated through training and experience with the intervention. In a different line of research, Stewart and Chambless [38] noted that provider attitudes about empirically-supported treatments (ESTs) could be improved by showing case studies along with clinical trial data. A similar approach may be worth investigating to increase LIT uptake. Our results also point to aspects of the patient perspective that would be helpful to highlight in future research and practice with the intent to increase LIT uptake, including the patient’s initial reaction to the LIT recommendation and their perception of the accessibility and appropriateness of the interventions.

## Data Availability

All data, code, and related materials are publicly available through Open Science Framework at https://osf.io/7hy2p/.
